# Significance of organizational health features during the COVID-19 pandemic for the well-being of Lithuanian healthcare workers

**DOI:** 10.3389/fpsyg.2023.1136762

**Published:** 2023-03-15

**Authors:** Milda Kukulskienė, Gita Argustaitė-Zailskienė, Aušra Griciūtė, Vilma Miglinė, Loreta Kubilienė, Nida Žemaitienė

**Affiliations:** ^1^Department of Health Psychology, Lithuanian University of Health Sciences, Kaunas, Lithuania; ^2^Department of Drug Technology and Social Pharmacy, Lithuanian University of Health Sciences, Kaunas, Lithuania

**Keywords:** healthcare workers, COVID-19, well-being, organizations, qualitative research

## Abstract

**Summary:**

During various emergencies, especially pandemics, there is a heavy burden on healthcare workers and pharmacists. Organizational support plays a significant role in protecting their mental health. Though the study aimed analyze the subjectively perceived difficulties and challenges of healthcare workers related to organizing work in the context of a pandemic.

**Methods:**

Twenty seven subjects (20 women, 7 men) participated in the qualitative research 30–45 min. Duration semi-structured interviews were performed, and thematic data analysis was applied.

**Results:**

During the first wave of the COVD-19 pandemic, research participants experienced an avalanche of change in all significant areas of life: experienced general overall uncertainty, confusion in working order, and intense changes in work functions, responsibilities, and workload. These changes reduced the scope for control and predictability, there was a lack of structure and clarity. The avalanche of change caused by the COVID-19 pandemic provoked a strong and controversial emotional response. The contradiction was revealed between helplessness, disruption, loss of control experienced by staff and the internal and external pressure to adapt as quickly as possible to the functions of caregivers. The threats posed by the pandemic reinforced the need for active and engaged leadership and highlighted the key features of an employee supporting organization.

**Conclusion:**

Surviving the avalanche of change caused by the pandemic, healthcare workers and pharmacists emphasized the importance of management decisions about managing patient and employee health threats, clear work organization, active and inclusive leadership, change planning, and organizational concern for employee sustainability and emotional well-being. Regular, systematic, clear and understandable, timely, open and sincere, uncontroversial, and consistent communication of administration provides security for employees and can contribute to better physical and psychological well-being of employees.

## Introduction

Crises, catastrophes, and disasters are constant threats that accompany life. Natural or manmade, such emergencies may elicit personal reactions and cause changes in the community. There is a growing body of evidence that the longer and stronger a person is exposed to stressors during an emergency, the greater the likelihood of developing mental health disorders will become (Lock et al., 2012). Possible consequences to a survivor’s well-being include mental health problems (such as PTSD, major depression disorder, non-specific distress, and others), physical health problems and concerns (e.g., self-reported somatic complaints or sleep disruption) and struggling with secondary emergency-related stressors like troubled interpersonal relationships or occupational stress (Norris et al., 2002). The researchers point out that social isolation and other changes caused by the pandemic may have affected people’s quality of rest and even their nature of dreams (Margherita et al., 2022; Monaco et al., 2022).

Though rise in distress was documented among the general population, healthcare professionals played a key role and were in a more vulnerable position during COVID-19: findings confirm that many of them are experiencing symptoms of depression, anxiety, insomnia and distress, with those working directly with COVID-19 patients at particular risk (Kinman et al., 2020; Olson et al., 2020). Other studies showed that during the COVID-19 outbreak considerable percentage of healthcare workers experienced mental disorders (Vizheh et al., 2020) and in the sample of various specialties healthcare worker burnout, anxiety and depression were actual burdens (Denning et al., 2021). In addition to personal challenges, the community infrastructure and a general sense of security may also suffer (Rodriguez et al., 2006).

In most cases, a person’s reaction to an emergency follows a common pattern. However, depending on many personal and societal factors, ranging from the severity of experienced life threat and loss of jobs to evacuation policies and public security, every emergency has its own differences (Nomura et al., 2016). Hamouche (2020) outlines five main stressors which are characteristic of the COVID-19 pandemic: (1) Perception of threat to safety, (2) Excessive information in the news and media, (3) Confinement and isolation, (4) Financial loss and job insecurity, and (5) Stigma and rejection in cases of infection. The pandemic is a unique situation and these stressors together with all that is yet unknown and unfolding warrant further research.

During crises and catastrophes, people are exposed to upheaval not only as individual beings but also often as a part of an organizational structure. An organization facing a crisis is presented with a particular challenge of helping its employees cope and maintain a sense of well-being, as they experience the situation from two roles: individual and that of a staff member.

At any point in an organization’s existence, the well-being of its employees should be one of its most important goals. Employee well-being may also contribute to a better financial state of the organization (Verwijmeren and Derwall, 2010), fewer unscheduled absences, less short-term disability leave, and better retention (Sears et al., 2013). According to Fredrickson’s (2001) Broaden-and-build theory, employee well-being may facilitate their novel ideas and awareness *via* the mediating effect of positive emotions.

Research data confirms the vital role of the organization on an employee’s health during the COVID-19 pandemic (Preti et al., 2020). A study by Kubilienė et al. (2021) pointed out that organizational factors supporting safety and stability may have acted as elements protective of physical and psychological well-being during the first wave of the COVID-19 lockdown among healthcare and pharmacy workers. Administrative staff have a key role to play in monitoring and supporting the mental health of their staff (Kinman et al., 2020). Shanafelt T. D. et al. (2020) analyzed concerns of healthcare professionals of all types during the pandemic and presented a set of requests that most employees have for their organizations in a pandemic situation: Support me, Protect me, Hear me, Care for me, and Prepare me. These requests have a parallel with Antonovsky’s construct of sense of coherence (SOC) which is a generalized resilience resource that manifests in three dimensions: meaningfulness, comprehensibility, and manageability (Stoyanova and Stoyanov, 2021). In a recent Italian study, a sense of coherence was positively associated with psychological well-being, confirming its critical role in helping individuals cope with stressors and traumatic experiences also in the context of the COVID-19 pandemic (Barni et al., 2020). In a recent Bulgarian study of healthcare professionals, SOC was validated as a possible determinant to predict the reduction of exhaustion and depersonalization as well as high levels of professional performance (Stoyanova and Stoyanov, 2021). SOC is known to be associated with an active adaptation through the use of generalized and specific resistance resources to avoid burnout in stressful situations (Antonovsky et al., 2021).

Different theories of emergency management stress planning, proper coordination, collaboration and learning as important contributions to the proper handling of emergencies and integration of psychosocial interventions (Migline, 2019). It was observed more than thirty years ago that poor planning can lead to poor management actions (Quarantelli, 1985). The author emphasizes that effective emergency management does not automatically arise from planning. Research shows that successful management depends on the organization of the communication process, leadership and coordination (Quarantelli, 1985). Appropriate delegation of roles and training of personnel in acquiring skills and knowledge is one possible action to diminish the negative pandemic impact on employees’ mental health (Giorgi et al., 2020), which leads to appropriate emergency management actions at all stages of the emergency management cycle, which is crucial for effective societal psychological resilience (Walsh et al., 2012).

Researchers note the need to develop a new approach and seek preventive measures to address occupational challenges in the work environment and reduce their negative impact on professionals (Molnar et al., 2017). It is important to see the issues of complex management of the COVID-19 pandemic situation and make appropriate decisions, not only from the perspective of an organization’s emergency management but also from the perspective of healthcare professionals, considering their unique experiences. De Kock et al. (2021) in a systematic review of 24 studies, most of which were conducted in urban hospitals, reported, that in resilience-building programs it is important to evaluate occupational and environmental factors. The more detailed the analysis and understanding of how healthcare professionals perceive, experience and behave under the effect of the ongoing COVID-19 pandemic situation, the more accurately healthcare organizations will be able to use specific insights for preserving the well-being and mental health of employees in similarly difficult situations in the future.

The aim of the study was to analyze subjective pandemic-related difficulties and challenges in work organization according to healthcare staff. The focus of the research was on understanding how healthcare professionals experienced daily life, well-being, work activities and management during multiple changes in organizational and individual practice in this unexpected and unique situation.

## Materials and methods

The article presents research data from the project “COVID-19 Pandemic-related Challenges, Psychological Well-being and Support Needs of Healthcare Workers and Pharmacists” (Funded by the Research Council of Lithuania, grant No. P-COV-20-44), the aim of which was to assess the physical and psychological well-being of healthcare workers and pharmacy specialists, as well as the challenges of work organization and assistance needs related to the pandemic caused by the coronavirus COVID-19. This article analyzes part of the project’s data related to the aspects of work organization and employees’ well-being.

To reveal the phenomena under research in-depth, qualitative research was chosen. The aim was to reveal the subjective experiences of healthcare workers and pharmacists in the pandemic situation, the difficulties and challenges of work organization related to the pandemic, the need for help and its availability during the pandemic. Qualitative research was chosen to gain a deeper understanding of the experiences of doctors, nurses, and pharmacists from an individual perspective (Creswell, 2003; Creswell et al., 2011). An inductive research strategy was applied, therefore, preconceived hypotheses were not formulated. Hypotheses were replaced with 2 open-ended and broad-based research questions: (1) What were the most common subjective experiences and challenges of work organization of healthcare workers and pharmacists in the pandemic situation? (2) What are the most relevant needs for help and how do research participants subjectively evaluate its’ availability in the pandemic context?

The study was conducted with the approval of the Kaunas Regional Biomedical Research Ethics Committee (No. BE-2-88; 20 July 2020). The data was collected remotely to enable the safe participation of employees of healthcare institutions and pharmacies throughout Lithuania and in compliance with the security requirements of the pandemic. Qualitative interviews were collected from 19 August to 24 September 2020. Doctors of various specializations, nurses, representatives of the administration of healthcare institutions and other employees and pharmacists were invited to participate in the research. Representatives of 222 healthcare institutions and pharmacy administrations were contacted and, following ethical requirements, an invitation to the interview was sent to the institutional e-mail addresses of the employees. To obtain a sufficient sample, the invitation to participate in the research was resent one more time. In the selection of the participants in the qualitative research, the aim was to purposefully include different cases, so the *snowball* selection method was used in parallel. The inclusion criteria were: the person was a healthcare worker (physician, resident physician or nurse), pharmacist and/or an administration representative, who worked in a public or private healthcare institution or pharmacy during the pandemic and gave a voluntary consent to participate in a study. Technical workers, cleaners, psychologists, social workers and unemployed people were not included.

The above-mentioned people participated in the research by contacting the responsible representative of the research and expressing their wish to participate in the research. Potential participants were introduced to the aim of the research, questions were answered, and the time and conditions of the interview were agreed on. Individual semi-structured interviews were conducted remotely, on an online platform suggested by the research participant (e.g., Zoom, MS Teams, or other) and lasted approximately 30–45 min. At the beginning of the interview, the aim was to establish psychological contact with the participant so that he/she, in a secure environment of mutual trust, could recall and discuss the experiences of the pandemic situation, the difficulties and challenges of work organization related to the pandemic, the need for help and its availability during the pandemic. All research participants signed the informed consent form electronically or live and agreed to record the audio of the interviews. The participants were asked not to mention personality identifying information (names, institution names, etc.) and if mentioned by chance, this information was anonymized and not included in the transcript.

The research involved 27 participants (20 women, and 7 men). The age of the research participants ranged from 24 to 63, with an average age of 39.82. The research involved 10 doctors (7 women, 3 men), 6 nurses (all women), 6 pharmacists (3 women, 3 men) and 5 administrative staff (4 women, 1 man). According to the status of a healthcare institution, 19 research participants worked in public and 8 in private institutions. More detailed information regarding demographic characteristics of the sample is presented in the appendices (see Appendix, Table A1).

### Data collection methods

The semi-structured interview method was chosen, which helps the researcher to maintain the structure and focus of the interview, but at the same time leaves room for revealing the side and unique aspects of the topic and the subjective experiences of each research participant.

The questionnaire was developed by a group of researchers. First, research participants were asked questions about demographic characteristics such as gender, age, type of workplace (public, private), and position (doctor, nurse, pharmacist, administrative employee, other position). The data were collected by 8 researchers of the research project team. The qualitative interview questionnaire consisted of key and refinement questions. The key questions are open-ended questions that do not point in the direction of a possible answer. They were asked all research participants, maintaining the same presentation structure. Clarifying, supplementary questions were asked only after the key open-ended questions to further saturate the responses of the research participants and to structure the content a little more. After the first two pilot interviews, the questionnaire was re-validated at an expert group meeting, with minor corrections. As the structure of the questionnaire did not change significantly, it was decided to include the pilot interviews in the final database as well.

1. Transcription

All the material collected during the 27 interviews was first transcribed to make it anonymous, but without distorting or altering the original language. The texts are written in the style of dialog between an experienced expert and a researcher. At the end of the transcription phase of the interview, a separate document was generated for each research participant, as well as an anonymized code for each document was given.

1. Qualitative data analysis methods.

The method of inductive thematic analysis according to V. Braun and V. Clarke was used for the analysis of qualitative data (Braun and Clarke, 2006. This method is one of the most widely used methods of qualitative analysis in the world. This method is described as universal and used to analyze a variety of ambiguous problems. The method of thematic analysis is quite flexible, as both external and deeper aspects of the research problem can be analyzed simultaneously (Braun and Clarke, 2006). Although the researcher is left free to choose specific research steps and methods, it should be emphasized that the methodological justification of this method is also strong (Creswell et al., 2007). Data analysis was obtained by applying 6 data analysis phases according to Braun and Clarke (2006):

1. Familiarizing with the data. This analysis stage included transcribing the data, reading the transcript, and noting initial insights in the researcher’s diary. The transcripts of each research participant were carefully read several times to understand the narrative as a whole.
2. Generating initial codes. Open inductive coding was applied. Firstly, the text of the interviews was divided into individual segments, which were then coded. The coding was performed in two stages: first, the words or compounds that make up the key phrases in the headings were extracted and coded. In the second phase, thematic categories were sought to group the already created categories into main categories. The analysis at the semantic and latent levels involved code classification, rearrangement, and assignment of sub-themes and themes. There were 314 pages of transcribed and encoded text in total.
3. Searching for themes. Recurring codes were collated into potential thematic units. At this stage, the researchers worked with excerpts from the text in a single document to combine the individual codes into common themes. The statements of the research participants, which are closely related to each other by logical, semantic and thematic connections, were grouped into categories.
4. Reviewing primary themes. At this stage, the generated codes were carefully checked many times, when new ways of grouping or separating them were sought. Themes were checked, changes in the formulation and meaning were made, depending on the code and the quote. The thematic model was generated.
5. Defining and naming themes. Specifics and definitions of each theme were discerned. Illustrative quotes and key features of each theme were described in a separate table. The coherent order of the themes was laid out. The validity criteria for external heterogeneity and internal homogeneity were applied. The thematic model was validated by methodological experts.
6. Producing the report. Clear, vivid, and the most substantive quotations were selected for illustrating the analysis. The structure of the thematic model was substantiated, and a written report of the analysis was produced.

## Results

**Figure 1:**
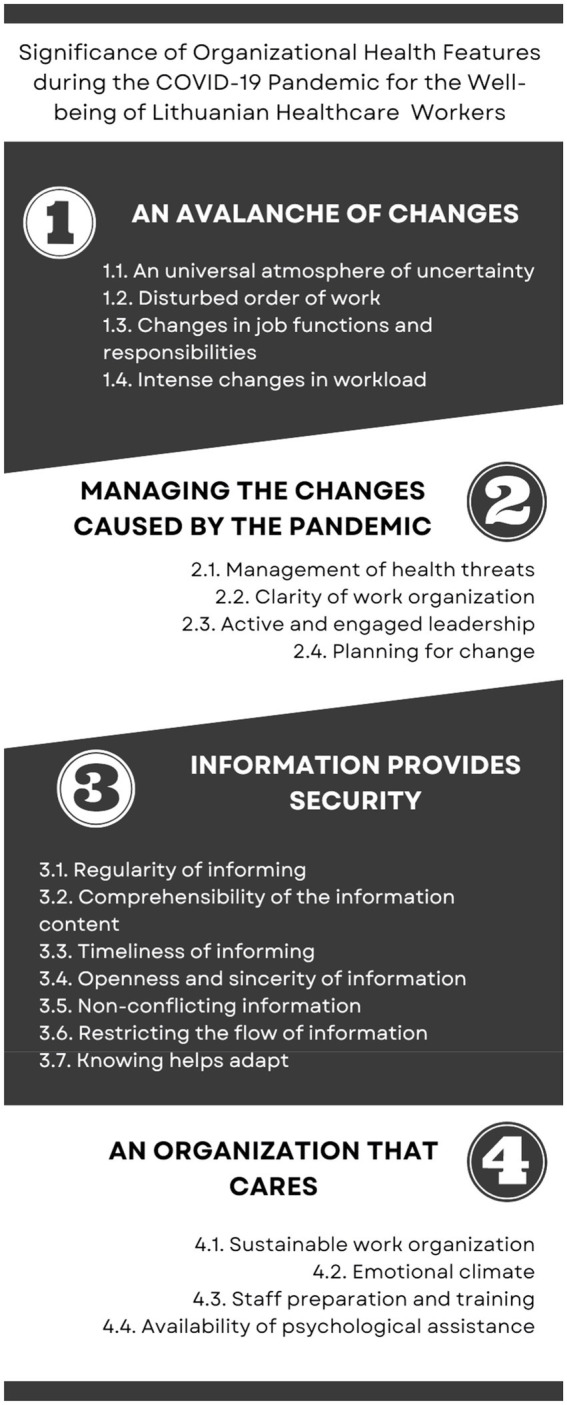
Map of themes and sub-themes.

The analysis of the semi-structured interviews with healthcare and pharmacists highlighted four main themes: 1. Avalanche of change (23); 2. Managing the changes caused by the pandemic (19); 3. Information provides security (16); 4. An organization that cares (27). These themes and 19 sub-themes are shown in the scheme of themes (see Figure 1). The number in parentheses after the title of each theme or sub-theme indicates the information about the frequency of each unit (in how many cases from 27 it was mentioned). The results reveal the attitudes of employees toward work organization processes during the first wave of the COVID-19 pandemic. It also describes the subjective experiences and needs of employees in coping with the changes in the work environment caused by a pandemic. Each theme and its sub-themes are explained in more detail, illustrated with quotes from the research participants’ language. At the end of the quotation, the code assigned to the participants is marked. Near the description of the sub-themes, the number of research participants in whose speech this sub-theme was highlighted is given in parentheses.

### An avalanche of changes

The start of the COVID-19 pandemic was revealed in the narratives of the research participants as a difficult-to-cover flow of change in all spheres of life caused by the threatening, uncertain, and global COVID-19 pandemic situation. The first theme describes in more detail the changes brought about by the start of the pandemic and the emotional response of research participants to them.

The sub-theme **An universal atmosphere of uncertainty** (23) describes the uncertainty experienced by research participants in a changed and unpredictable daily routine. At the start of the pandemic, the COVID-19 virus was new and unknown, and life became difficult to predict and full of uncertainty: *“Well, that’s it now: you do not know if you are going to get up healthy or not tomorrow, whether you will have a fever, will they let you work or not”* (13-S). For some research participants, this uncertainty was particularly difficult to bear: *“*<... > *uncertainty seems to cover all areas of your life. If it was in only one [sphere], well, then somehow. And here it is everything: family, relatives, and the work itself, and the patients. Somehow this was very difficult.”* (7-G). The universality and generality of the uncertainty during the first wave of the pandemic inevitably affected all experiences in the work environment.

Another sub-theme **Disturbed order of work** (22) reveals employees’ experiences in a changing and unclear work order. Confusion prevailed, and research participants felt deprived of a solid basis and usual clarity. There were many difficulties with the new requirements and work order, but it was difficult to find the answers: *“*<... > *nobody [knows] anything - everyone is walking around and does not know how, so what do we have to do here - what do not have to do, so what do we have to do here now?* (9-G)*.”* The participants lacked information, structure, and clear guidelines and instructions on how to work: *“How do you know how to take good care of them [patients]? Because that information, especially at the very beginning, changed quickly, there were no approved guidelines of how and what to do here.”* (4-G). The confusion was intensified by the lack of firm decisions from senior management: *“How will it be here, how to organize ourselves here, there were no such firm decisions by the ministry on how you have to behave.”* (25-AG).

**Changes in job functions and responsibilities** (21) took place when transitioning from contact to remote work, at the beginning of active testing of hospitalized patients, when the necessity for disinfection of premises, the use of protective equipment and technical management of flows increased, etc. Staff turnover was very intense in some departments: *“Our department is made up of all the departments in the hospital, just today I calculated that at the moment my team is made up of people from 14 departments, so really all people are with different experiences, with different moods, with different habits of some sort.”* (26-AS). The redistribution of staff posed a variety of emotional challenges and intimidated: *“Not the fear of the disease itself, but the fear of what, what patients we will receive. What will you need to do with them?”* (15-S). Staff had to adapt to the work of the new department each time: *“When I found myself working in a different department for a month, and ... Only then did I really realize that it was emotionally exhausting.”* (14-S).

Nearly half of the survey participants talked about the **Intense changes in workload** (21). The increased workload was due to the testing of hospitalized patients, the use of protective equipment, and other, at first glance, small but time-consuming tasks. Work in pharmacies also intensified: *“Of course, the queues. The working time was automatically extended significantly as the service time per person was much longer, with all the disinfections and with everything. Then, clearly when the purchasing of drug stocks began* < ...>*”* (17-F). Administration staff mentioned that they often had to work overtime: *“Until we settled everything and put it together, we did not have enough time to go to the toilet, we worked in three shifts. Well, you spend a lot of time at work.”* However, some employees, by contrast, spoke of a reduced workload. According to these participants, this was due to the extremely cautious public reaction to the first quarantine and the delay to contact doctors for fear of catching COVID-19 in healthcare institutions: *“*<... > *that anxiety of coronavirus overshadowed < ... > the whole situation and they [patients] were not there then”* (4-G). Strong inconsistencies in participants’ workload estimates suggest an overall imbalance in workloads: some resources became obsolete or underused, others were used in excess of normal workloads.

In summary, during the first wave of the COVID-19 pandemic, research participants faced an avalanche of change in all significant areas of life: experienced general universal uncertainty, confusion and puzzlement about work procedures, intense changes in work functions, responsibilities, and workload. These changes reduced the scope for control and predictability, there was a lack of structure and clarity. The avalanche of change provoked a strong emotional response and revealed the contradiction between helplessness, confusion, loss of control of the employees and the internal and external pressure to adapt to the changes as quickly as possible, to perform the functions of the caregivers properly.

### Managing the changes caused by the pandemic

The challenges and efforts required to manage the avalanche of change caused by the first wave of the COVID-19 pandemic have emerged as a crucial aspect of this research. This is covered in the second theme, reflecting the research participants’ insights into managing the challenges of the first wave of pandemics in organizations. This part describes the barriers and good practices in change management perceived by the participants and reveals staff expectations and suggestions for administrations to successfully manage the challenges of COVID-19 and other emergencies in the future.

The first sub-theme was **Management of health threats** (19). Maintaining the physical health of patients and staff emerged as a prime and top priority for research participants: *“That clear distinction made between those more dangerous patients and those less dangerous, less likely to have the virus certainly provided security.* <...>*”* (4-G). Participants welcomed the opportunity for older employees to work in departments where it was easier to ensure safer working conditions. On the other hand, the inflexibility of the decision to work remotely and the lack of exemptions have been criticized: *“*<… > *It is wise that there is the possibility of both remote and live consultations. <... > When contact is forbidden, let us say, it’s definitely not good and healthy.”* (10-G). It was important for the workers that these decisions were weighed. Pharmacists placed particular emphasis on the need to provide workers with adequate protective equipment: *“Many challenges arose < ... > at the beginning there was a clear lack of masks and protection, and there was no glass at the checkout. Am... I even had myself, until the worker brought and installed it, I even had to use self-made protection.”* (20-F). Participants of the research also mentioned the lack of decisive organizational solutions for disinfection and ventilation of the premises, ensuring the regular testing of employees, rapid supply of goods and work equipment such as medicines and technical equipment, and personalized solutions.

Some research participants rated the management of the pandemic changes in their workplace positively. In these cases, **Clarity of work organization** (12) was of great importance: *“There were very clear instructions from the management. <... > Our pharmacy was definitely protected. <... > Maybe in this case I felt safe at work. In the sense that everything is already done.”* (17-F). Clear and firm decisions by the organization increased the sense of control of the situation: *“<... > it gave certain structure, more, in a sense, more time, did not it, less hurry and more structure for each case.”* (2-G). This gave employees the opportunity to feel better in the work environment, increased satisfaction, and likely motivated them to do their job better. On the contrary, another part of the research participants, such as administrative staff, stated that there was a lack of clarity in work organization in their institution: *“How will it be here, how to organize ourselves, there were no such firm decisions by the ministry on how you have to behave.”* (25-AG).

During the pandemic, the need for **Active and engaged leadership** (12) in organizations increased. It was important for the staff that the head of the institution was visible and accessible: *“When we have a prominent leader, let us say, a nursing administrator, well, maybe you know, she helps us „pay attention to this, it is very important here”* (13-S). The importance of flexibility and active response were also mentioned. Lack of leadership increased feelings of insecurity and frustration, participants were annoyed by uncertainty, lack of quick decisions and instructions. This increased disagreements and confusion within the team, distrust of management and their decisions: *“Well, a lot of that, let us say, everyone is talking, everyone is screaming and, oh, and there is no commander [laughs]”* (9-G). Employees also mentioned a lack of responsibility and support from management: *“<... > [there was a lack of] responsibility from our clinic manager and both better communication and better support in, let us say, this situation, more responsibility.”* (5-G). In some cases, the problem of insufficient understanding of the practical work of the institution’s administration was also mentioned: *“The administration should come to the department more and spend some time. Not to check how we work, but to spend some time. Be there, and feel what kind of work it is, how everything is going on”* (14-S).

Another sub-theme was **Planning for change** (11). The research revealed that participants lacked preparation for a pandemic situation in the work environment: organizations lacked security measures, uncertainty and confusion prevailed, and decisions on work procedures were often made chaotically: *“It changed a lot, apparently all SAM [Ministry of Health] and the whole government were not fully prepared”* (14-S). Employees emphasized the need not only to have a vision but also to focus on preparing for the implementation of the change plan strategy: *“We saw that there was not only plan B but also plan C and plan D, that preparations had been made for more complex scenarios which were not needed.”* (4-G). Some participants of the research felt a severe lack of calm, objective, non-emotional planning for change. It has been mentioned that many things can be foreseen and prepared for. As an example, it can be mentioned that not only the transition to emergency mode, but also the return to a normal work order can be emotionally difficult and cause difficulties: *“this period when we suddenly [sighs] had to regroup from the quarantine regime to almost no quarantine regime, it also caused anxiety and confusion among the team, and for me personally, amm ... there was a little anger at the administration”* (1-G).

In summary, managing the change caused by the pandemic has become a complex challenge in many healthcare and pharmaceutical institutions. The threats posed by the pandemic strengthened the need for active and engaged leadership. Employees emphasized the need for clear work organization, purposeful and flexible leadership, change strategies and planning.

### Information provides security

The third theme reveals the peculiarities of the organization’s internal communication with employees. This theme consists of sub-themes reflecting communication trends in the workplace during the first wave of the COVID-19 pandemic. This section will discuss employees’ attitudes toward how the administration and management communicated with employees about change management measures and decisions. It also sought to understand how employees were subjectively affected by administrative communication and what communication they expected.

Participants highlighted the need for **Regularity of informing** (10) in their organizations. Information was provided regularly and continuously in the workplaces of some of the research participants: *“[management’s instructions] came every day with updates on what we should and should not be doing.”* (17-F). In these cases the administration regularly communicated in various ways: through e-mails, calls, live chats. Regular, uninterrupted information was positively assessed and created a sense of security. On the contrary, several participants mentioned a lack of information, stated that communication with management was too infrequent and that there was a lack of structured, objective information: *“I would like this quiet planning. You do not need that whole panic. So much of that negative information. You need to be calm, inform objectively and work while planning. And not suddenly run around and scare everyone.”* (23-AG).

Furthermore, employees positively assed the **Comprehensibility of the information content** (16). During the first wave of the pandemic, the confusion and insecurity of employees were exacerbated by ambiguous information. Uncertainty was exacerbated by the fact that the information transmitted by the administration was not selected and adapted to specific jobs or groups of employees: *“<... > information from the administration [unclear words] is very broad, very scientific, and in the end, you read, read, read, read, really and forget, and, well, how to say, it’s too complicated.”* (16-S). It was important for employees that management selected information and adapted it: *“we want more clarity, especially from the higher governance, so to speak, our structures. Yes. Well, and [that information] would be clearer to the ordinary person. Who is not a lawyer, who is not some kind of specialist.”* (3-G). There was also a need to explain orally the written information provided to the staff: *“<... > [the laws] might be somehow, I do not even know, presented perhaps in more detail. Not just sent in the email.”* (7-G).

**Timeliness of informing** (13) was also important. A significant number of research participants mentioned that important information reached them late: *“We doctors, as you know, all work according to norms. <... > Well, out of all of these, it was probably only at the end of May that they[norms] were prepared, only practically after the quarantine.”* (27-AS). In the absence of timely information, rumors spread in the institutions: *“As you say, everything is happening, as I say, in the form of ‘rumors’. That’s, well, that’s bad.”* (16-S). On the contrary, timely administration communication reduced staff confusion and provided reassurance: *“<... > there were timely decisions that, well, gave the workers such peace of mind”* (2-G).

The research showed that workers expected the **Openness and sincerity of information** (7) and wishedto know the truth about the real situation within the organization. As the doctor said, she wanted *“more humane communication with subordinates on the management’s part.”* (9-G). They also mentioned that it is important not to forget the encouraging and inspiring information. In the internal communication, the participants lacked the felt support of the management and gratitude for their work: *“<... > well, and that it is not only thanking people but also showing others that it is nice that people have taken responsibility and done something. That’s very good, we are happy about that.” (*5-G). Fake, exaggerated optimism and the desire to cover up problems were also viewed with skepticism. Perceived omission or even concealment of information reduced employees’ confidence in the information received and increased insecurity.

The research participants lacked for **Non-conflicting information** (8): *“At that time, practically every day there was something new to be changed, annulled, so that was really hard”* (7-G). Contradictory, inconsistent information increased distrust of management, raised insecurities: *“Well, that was a constant ambiguity, because anyway, a lot of people blamed the management, the local management of the polyclinic, we all did not know how to behave, and there were changes.”* (8-G). In internal communication, research participants lacked consistency: *“<…. > chaos, when one day it is said to do things one way, the next day it is quite the opposite. One day to do this - the next day this is already bad.”* (10-G).

The research participants confronted a huge amount of information, which they described as endless and they recommended **Restricting the flow of information** (12). Too much new information that is difficult to cover created tensions and challenges: *“At the very beginning, when many different commandments began to come, it was very difficult. High flow of information and [difficult] to understand. Everything would change abruptly, almost hourly. Well, it was not easy to deal with such very large flow of information”* (13-S). Excess information was demotivating. It was also annoying that the flow of information was unmanageable: *“You get that letter like at 11 in the evening, because then the manager or deputy manager sums up all the events of the day, and you are already asleep, when tink-tink, the phone rings.”* (1-G). A significant number of research participants talked about the fact that they regularly received e-mails and received phone calls from the administration after work or in their free time. It made them constantly think about the pandemic and work and did not allow them to rest from work qualitatively.

The research participants emphasized that **Knowing helps adapt** (9). Knowing created a sense of security and confidence in the work environment: *“It would be much safer and, as one says, more confident, if you heard it from them, well, here’s what you need to do. Because these are, in fact, the people who control everything and you trust them, much depends on them.” (*16-S). Over time, the gained knowledge and practical experience helped the employees to adapt: *“*<... > *during those months we already got used to it, [laughs] and we already know the order, <... > it no longer causes that unstable state* < ...>*”* (5-G). The research revealed that knowing not only improved adaptability but also reduced stress: *“<... > at the beginning there was that uncertainty, and then when we saw that the [personal protection] means are sufficient, some information appeared on how to deal with it, when we saw that there were not too many of those patients, so there was the feeling of a bit of control, that everything is not bad here so far.”* (4-G).

The research showed that regular, systematic, clear, understandable, timely, open, sincere, uncontroversial and consistent communication with employees can contribute to more successful employee adaptation and well-being. To organize work more smoothly during a pandemic, it is necessary to manage, select and limit the flow of information sent to employees. This saves employees and helps prevent information fatigue.

### An organization that cares

The fourth theme combines sub-themes about the efforts of the organizations of healthcare workers and pharmacists to take care of the well-being and psychological needs of employees. This sub-theme includes not only employee-friendly work organization but also the attention paid by the management of organizations to the psychological atmosphere in work teams, the solution of emotional challenges, and the focus on appropriate employee preparation and psychological well-being.

A significant number of the research participants emphasized the need for **Sustainable work organization** (13). In situations such as the pandemic caused by COVID-19, it is necessary not only to organize work smoothly, but also to take care of staff sustainability: *“If the workload is inadequate, you get too tired and then mistakes can start, and then you can say a ruder word to the patient, so yeah, so that the workload was adequate, so you could work and did not need to rush and everything would smoothly and you would not be called to three or four wards at a time < ...>”* (13-S). Excessive workload and intensity negatively affected the quality of work and increased the risk of errors and burnout. Participants were pleased that in some cases there was an adequate response to staff shortages and appropriate decisions were made: *“Over time, additional staffing has been implemented to ensure that the ideas on paper are realized on a day-to-day basis.”* (4-G). The research participants also highlighted the need for more frequent breaks during work; they helped them to take care of work safety (ventilate the premises, carry out disinfection) and enabled them to recover physically and emotionally: *“Breaks also helped to get away from work for a short time, to restore psychological balance: At least for half an hour to take a breath, I’d say, both to go to the toilet and have a drink.”* (15-S). The research participants assessed the opportunities for communication with the administration “bottom-up” positively: to express criticism, to consult, to ask, to be supervised. It reduced the feelings of exclusion and helplessness: *“If it is really totally difficult or there are some difficulties, then we can contact the head of the region … We were not left alone to deal with some problem*…*”* (17-F). In cases where the organization lacked attention to employee feedback, they felt unheard and not listened to.

Experienced “teamwork” and peer support were significant protective factors in facilitating the **Emotional climate** (18). The participants spoke emotionally and warmly about the team’s focus and support: *“In fact, there was also a lot of support from our newly formed team, <... > but the team then just then grabbed me, shook me and would say “we do everything here by ourselves, too” so like, “you just stay close.” And you recover, and you start again.*” (26-AS). Several research participants mentioned that the changes caused by the pandemic even led the team to become more focused: *“It turned out to be a good team and understanding and working conditions and everything... Well, and support that we have to work on the front line and we do work. This kind of mobilization in the event of a disaster”* (18-F). On the other hand, almost half of the specialists who participated in the research talked about the escalation of tensions in the team: *“Of course, I raised my voice at her, she started crying [laughs]– it happened when we were on call – in that sense, such things were just happening because there was no system.”* (9-G). In some cases, tension intensified because of the high intensity of work, staff shortages and tensions caused by some colleagues becoming infected with COVID-19. Pressure from the administration and unfounded accusations against the employees did not help to solve the difficulties: *“There were many difficult patients and < ... > the staff began to fall ill. And then it was hard, because then all sorts of things began, not support, but more like witch hunts. Why, what are you doing wrong, what are your schedules, how are you getting dressed?”* (23-AG).

One of the most important goals set by healthcare workers and pharmacists during the first quarantine was **Staff preparation and training** (9). There was a particular need to train and prepare staff for departmental restructuring or assigning new work functions: *“Even, let us say, working with infectious patients – we were completely unprepared. And, and we really wanted a little training.”* (16-S). The research participants emphasized that to place high demands on specialists, it is necessary to invest in their proper training: *“So that there would be time to train everyone, that job, that there would be no such stress, if you want the specialist to be serious, it is necessary to have the training*.” (14-S). It is a long-term investment that contributes to the well-being and psychological resilience of healthcare specialists.

One of the most saturated sub-themes was the **Availability of psychological assistance** (27). For some employees, the first quarantine period was a particularly difficult time requiring the help of a mental health professional: *“That kind of, really, professional help was needed, I think it would not have been amiss. It did not seem necessary at the time, but now when you look back ... you realize it would really have been very, very helpful.”* (10-G). The majority of healthcare workers and pharmacists who participated in the research were positive about the availability of psychological help in their environment. However, several research participants indicated that the availability of psychological help in their work environment was not ensured: *“T: And tell me, how do you rate the availability of psychological help in your environment? D: Zero. Zero. There is none.”* (9-G). It was also mentioned that psychological help in the work environment for employees may be unacceptable and, in some cases, it would be safer to seek help outside: *“*<... > *I would not dare go to my institution myself. Just, there would be some kind of barrier and that’s it. Like going to a classmate, or a friend of a friend. Well, you want a bit of a distance.”* (8-G).

In summary, the analysis of the research data revealed the importance of a sustainable work organization, the organization’s concern for the emotional climate of the team, proper staff training and ensuring the availability of psychological help to employees. The good emotional well-being of employees and the organizational climate that promotes the psychological resilience of employees are the basis for a successful organization which can overcome difficult challenges. Concern for the individual needs and emotional well-being of employees, maintaining a warm, open and peaceful emotional climate in the organization is a meaningful investment in the success and prosperity of the organization. Employee well-being is the real and most important capital of an organization.

## Discussion

Covid-19 pandemic required rapid and effective organizational change: decisions made at both state and internal organizational levels affected the effectiveness of pandemic management. Many studies on this topic point to the significant role of organizational culture in the management of the Covid-19 pandemic: organizational support, caring for its employees, promoting information, leadership, and mutual assistance are the organizational factors that help meet the challenges of the pandemic (Shanafelt T. et al., 2020; Kubilienė et al., 2021). The main aim of our research was to analyze subjective pandemic-related difficulties and challenges in work organization according to healthcare workers. It is crucial to understand how healthcare workers perceived and survived the COVID-19 situation - solutions based on original data can help organizations reduce the risks to the physical and emotional well-being and health of their employees.

In this research, we used a qualitative approach to data collection and analysis that provides a deeper insight into the healthcare workers’ experiences at the beginning of the COVID-19 pandemic and the first quarantine wave to understand their daily lives, well-being, participation in the work environment and unplanned organizational changes during that time. Our research broadens the understanding of pandemic management factors relevant at the organizational level and their potential interaction in the event of a pandemic. We identified 4 qualitative themes and mapped them, highlighting the most important organizational factors that emerged during the research.

The results of the research revealed several relevant themes. One of them was identified as an “Avalanche of Change” - uncertainty, confusion in the work order and the intense change of work functions, responsibilities and workload experienced by the participants. Especially at the beginning of the pandemic and the first quarantine, the participants lacked balance, structure, and clarity during these changes. These results echo other research which highlights that healthcare workers face significant challenges, high-stress conditions, and burnout syndrome during the COVID-19 pandemic. (Giusti et al., 2020; Kinman et al., 2020). Our results also complement and respond to the findings of other researchers with new insights into the contradiction between the helplessness, confusion, and loss of control experienced by staff and, at the same time, experiencing internal and external pressure to adapt to the changes as quickly as possible, and efforts to perform caregiver functions properly. It is known that if staff feel unsafe, it can cause symptoms of stress and burnout and reduce their ability to work effectively. These symptoms can morph into post-traumatic stress disorder or other chronic diseases (Wu et al., 2020). Conversely, for healthcare workers, a positive approach to a stressful situation can be an important protective factor (Babore et al., 2020).

In response to the chain of changes taking place, emergency management organized by the leaders has emerged as a critical aspect. In the theme “Managing the changes caused by the pandemic,” participants highlighted management decisions on managing threats to the health of patients and staff: protecting the physical health of patients and staff has emerged as a top priority for the participants. The need for clear work organization, active and inclusive leadership, change strategies, and planning has also emerged in this theme. Clear organizational decisions and systematic management of health threats had a positive effect on the functioning of employees in the work environment. Our insights echo the findings of other research that in a pandemic, the most important things are an action plan, advance preparation, and organizational support (Huffman et al., 2020). Our research identified the importance of learning lessons, having a vision, and focusing on preparing for the implementation of a change plan strategy, as clear preventive steps and preparation for different outcomes in a situation contribute to the effective functioning of employees in an organization. Other researchers also mention key aspects of crisis management, emphasizing a clear, optimistic vision and a realistic plan, decisive actions, open, honest and frequent communication, and gratitude to employees (Wu et al., 2020). We found that during the pandemic, there was a particularly high need for active and engaged leadership in organizations, which was seen as significant help. This result of ours also reflects the results of other research that emphasize that leaders need to understand and recognize the needs of healthcare workers (Ahmed et al., 2020; Shanafelt T. D. et al., 2020; Shanafelt T. et al., 2020; Wu et al., 2020).

Proper communication is one of the key strategies for crisis management. The third theme revealed in our research is on the peculiarities of the organization’s internal communication. It was found that regular, systematic, clear and understandable, timely, open and sincere, uncontroversial and consistent communication by the administration leads to better adaptation and well-being of employees and reduces stress. This message echoes comments from other researchers that information helps to reduce anxiety (Wu et al., 2020). Our research also highlighted the need expressed by participants to manage, select, and limit the flow of information to employees in the organization to avoid excessive exposure and information fatigue. These findings resonate with the insights of other researchers that problematic use of social media has been linked to stress and sleep difficulties (Lin et al., 2020; Que et al., 2020).

Theme “An organization that cares” combined sub-themes about the well-being needs of employees in the organization identified by healthcare workers and pharmacists. The research found the importance of sustainable work organization, the organization’s concern for the emotional climate among the staff, proper staff training and ensuring the availability of psychological assistance to employees. Giorgi et al. (2020) in the narrative review of 37 studies mentions the importance of safe work environments on employee’s psychological wellbeing in the presence of COVID-19 challenges. An employee who feels good and safe in the organization can do their job better and more attentively and learn new things faster (Sanner and Bunderson, 2015) The organizational climate that promotes the psychological resilience of employees predicts how the employee will value the organization and how much they will show solidarity with the values of the organization. These findings reflect the findings of other researchers. Leaders should anticipate that mental health problems in healthcare facilities are rising. Therefore, it is very important to identify this problem and establish support resources when building support teams (Wu et al., 2020).

### Advantages and applicability

One of the main advantages of this work is the large sample size, considering the specifics of qualitative studies (n = 27). The inductive method of analysis helped to analyze a large amount of textual material in a very detailed and non-template way. Working in a team of experts not only helped generate ideas for finding the most appropriate way to structure, formulate, and interpret codes, sub-themes, and themes, but also contributed to the greater validity of the qualitative analysis. The qualitative research strategy allowed for naturalistic research and interviewing of the first and one of the most vulnerable units during the pandemic – the providers of help. The results of this research provide comprehensive, in-depth insights into the needs of work organization in the context of emergencies. The results are of a recommendatory nature. They can be interesting and practical for organizations’ leaders and administrations to set priorities, identify problems, make more effective decisions in critical situations, improve the climate of organizations and sustainably take care of the well-being of employees. Descriptive data is richly illustrated with quotes and examples that can serve to generate new ideas and solutions. The publication may also be of interest to healthcare workers and pharmacists who feel the need to reflect on the experience of the pandemic and are looking for ideas on how to take care of their psychological well-being. The identified key themes and sub-themes can provide data for the formulation of new insights and hypotheses that can be tested in quantitative and qualitative research. From a scientific point of view, these data may also be valuable for the future development of job satisfaction scales or questionnaires. In future quantitative research, it would be valuable to objectively assess the identified categories. Repeated measurements and longitudinal or experimental research would help to assess well-being more objectively and to monitor changes in well-being and causal relationships in different contexts: various professional settings, evaluating various risk personal and societal factors (Giorgi et al., 2020; Vizheh et al., 2020; De Kock et al., 2021; Denning et al., 2021).

### Limitations

Electronic invitations and convenient selection of research participants may not guarantee representation of some health professionals’ groups in the study sample, because people who respond voluntarily may be more motivated, more active, or willing to share their experiences, which may differ from peoples’ perception and apprehensions who are unprepared, or unwilling to share their narratives. On the other hand, the voluntary response may have influenced the inclusion of participants who were more emotionally affected by the pandemic, e.g., feeling more pain, frustration or anger. It is also possible that some potential study participants use less or do not use electronic means of communication, and this may have deterred them from deciding to participate in the interview. Application of snowball sampling technique resulted in the study sample not being proportional in terms of gender and public/private sector representation. The results of our study are more characteristic of the female gender and professionals working in the public sector. Quantitative studies of representative samples could reduce these limitations. To avoid the risk of infection, semi-structured interviews were conducted remotely, through online communication means. However, psychological experience suggests that contact conversation allows to capture non-verbal information more precisely, to feel safer about the interpersonal contact, therefore, it can be expected that the conversation would be more open and comprehensive.

The methodological choice in this study was to obtain the research data that reflects the experience of healthcare professionals in various work fields. To gain a deeper understanding of the experience of some concrete profession, the narratives of representatives of the chosen profession should be analyzed. The data allowed us to identify four key themes (“avalanche of change,” “pandemic change management,” “security provides information” and “organization of concern”) and their sub-themes, and these findings help to form a deeper knowledge and more complete and comprehensive picture of research participants’ behaviors and emotions, but do not provide substantial new information and are consistent with data from other studies that examined the experience of healthcare professionals during COVID-19 quarantine. Qualitative research data provides valuable insights into the experience during the first wave of COVID-19 quarantine – obviously, the experience gained during other phases of an evolving COVID-19 pandemic may have its own specificities, therefore further investigations are required.

## Conclusion

1. Experiencing the avalanche of change caused by the pandemic, healthcare workers and pharmacists in the management of change emphasized the importance of leaders’ decisions about managing patient and employee health threats, clear work organization, active and inclusive leadership, change planning and organizational concern about employee sustainability and emotional well-being.
2. Regular, systematic, clear and comprehensible, timely, open and sincere, uncontroversial, consistent communication from the administration provides security for employees and can contribute to the better well-being of employees.

## Data availability statement

The raw data supporting the conclusions of this article will be made available by the authors, without undue reservation.

## Ethics statement

The studies involving human participants were reviewed and approved by Kaunas Regional Biomedical Research Ethics Committee. The patients/participants provided their written informed consent to participate in this study.

## Author contributions

AG, MK, VM, LK, and NŽ conceived and designed the study. AG, MK, and VM collected and prepared the data. MK, AG, and NŽ analyzed the data. GA, MK, AG, LK, VM, and NŽ wrote the manuscript. All authors contributed to the article and approved the submitted version.

## Funding

The article presents research data from the project “COVID-19 Pandemic-related Challenges, Psychological Well-being and Support Needs of Healthcare Workers and Pharmacists” (Funded by the Research Council of Lithuania, grant no. P-COV-20-44).

## Conflict of interest

The authors declare that the research was conducted in the absence of any commercial or financial relationships that could be construed as a potential conflict of interest.

## Publisher’s note

All claims expressed in this article are solely those of the authors and do not necessarily represent those of their affiliated organizations, or those of the publisher, the editors and the reviewers. Any product that may be evaluated in this article, or claim that may be made by its manufacturer, is not guaranteed or endorsed by the publisher.

## Appendix

**Table A1:** Demographic characteristics of the study sample.

**Gender (M/F)**	**Age (Years)**	**Organization (Private/public)**	**Position**
M	31	Public	Physician
F	47	Public	Nurse
M	46	Public	Administrative employee, physician
F	59	Public	Administrative employee, nurse
F	35	Public	Nurse
F	29	Private	Physician
M	28	Public	Resident physician
M	31	Public	Physician
F	27	Public	Resident physician
F	52	Public	Nurse
F	33	Public	Physician
F	31	Private	Physician
F	32	Public	Physician
F	63	Public	Nurse
F	50	Public	Administrative employee, physician
M	43	Private	Pharmacist
F	34	Private	Pharmacist
M	24	Private	Pharmacist
M	43	Private	Pharmacist
F	36	Private	Pharmacist
F	27	Private	Pharmacist
F	43	Public	Administrative employee, physician
F	46	Public	Administrative employee, nurse
F	45	Public	Nurse
F	59	Public	Physician
F	47	Public	Nurse
F	34	Public	Physician
